# Gabor’s holography at sea

**DOI:** 10.1038/s41377-019-0133-2

**Published:** 2019-01-30

**Authors:** Gabriel Popescu

**Affiliations:** Quantitative Light Imaging Laboratory, Department of Electrical and Computer Engineering, Beckman Institute for Advanced Science and Technology, University of Illinois at Urbana-Champaign, Urbana, IL 61801 USA

**Keywords:** Optical sensors, Imaging and sensing

**Fig. 1:**
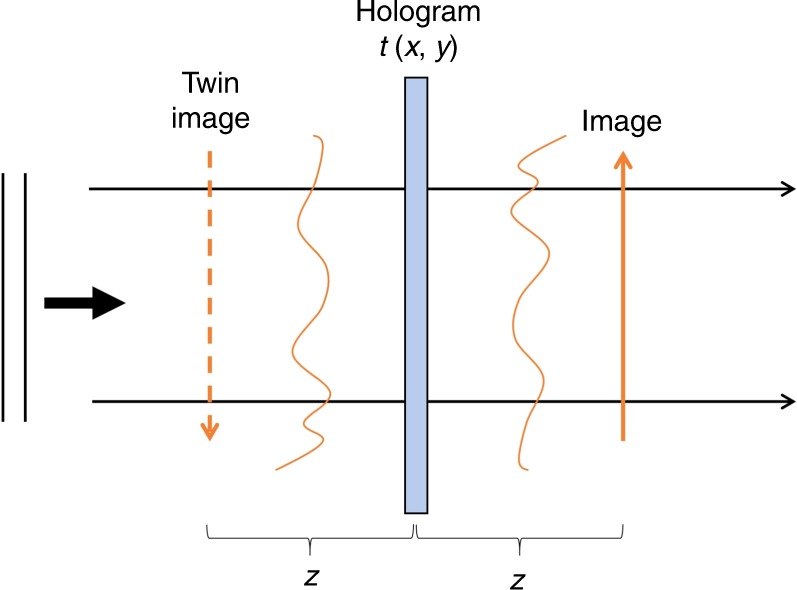
Reading a Gabor hologram generates a real and twin image overlaid along the optical axis. The distance *z* denotes how far the recording film is placed with respect to the object

In 1948, predating lasers by more than a decade, Gabor proposed a “new microscopic principle”, which was initially aimed at correcting spherical aberrations in electron microscopy^1^. While the method has not produced an impact in its originally intended field, it opened a new direction, known as *holography*, in optics. Gabor’s technique allows one to store phase information by recording on film the intensity of a field emerging at a certain distance from an object. The image of the object itself can then be reconstructed by shining the same incident light onto the film. Holography created an impact on the lay community, especially because parallax made the imaged objects appear “3D”. However, this type of *in-line* holography suffers from a major limitation: the in-focus image is always overlaid with an out-of-focus version of itself (see Fig. 1). The reason for this limitation is as follows: since the recorded signal is real (intensity signal), its Fourier transform is Hermitian, consisting of two complex-conjugated terms, namely, the *in-focus* and the *twin* image. This obstacle prevented holography from becoming a practical tool until Lohman, Leith, and Upatnieks reported *off-axis* holography, which shifts the twin image away from the optical axis^2^.

In recent years, combining phase-contrast microscopy and holography with digital detectors has yielded optical pathlength maps associated with transparent specimens, which has proven valuable in biomedical applications. Thus, *quantitative phase imaging* (*QPI*) provides a label-free imaging modality without the photoxicity and photobleaching associated with fluorescence. QPI solves the twin-image problem with either phase-shifting or off-axis interferometry (for a recent review, see ref. ^3^).

Ozcan and colleagues showed that *deep-learning* can be used to remove the twin-image from a single-intensity measurement, thus eliminating the need for specialized interferometers and multiple acquisitions^4^. This convolution neural network approach provides a fast means for phase image recovery for various cell specimens, including pap smears. Because the phase retrieval is entirely computational and the imaging system only requires an intensity measurement provided by a common detector, e.g., a CMOS camera from a mobile device, the method can be implemented in a highly compact, portable instrument. As a result, the Ozcan group recently has published a follow-up report describing such an instrument dedicated to high-throughput analysis of sea water samples^5^.

Plankton represent an important component of the marine ecosystem, and studying its composition is of high importance. Clearly, instruments dedicated to this problem must operate in the field at high acquisition rates to allow a significant quantity of water to be tested. An inline holographic system paired with machine learning, as reported by Göröcs et al.^5^, fulfills this challenge and enables the real-time testing of sea water at 100 ml/h. The system is battery powered by a laptop and extremely portable. Taking advantage of parallel (GPU) processing, the system can resolve 700 objects/ml and mitigate motion blur by using the color information available for each pixel.

These are very exciting times for artificial intelligence (AI). Initially driven by the immense amount of data generated by consumer products, machine-learning algorithms are now also opening new avenues in scientific applications. With large quantities of high-quality training data, AI is a powerful tool for segmentation and classification, which is likely to impact a range of biomedical fields, such as histopathology, radiology, genomics, and cell biology. Of course, AI poses some challenges as well. For example, many problems do not present unique solutions (such as the phase retrieval from intensity signals described above); thus, outputs must be constrained and validated by prior knowledge anchored in physical reality. However, pairing artificial intelligence with genuine (human) intelligence, as illustrated here^4,5^, is likely to become a widespread part of the scientific method in the future.
